# Relationship between body mass index and perceived insufficient sleep among U.S. adults: an analysis of 2008 BRFSS data

**DOI:** 10.1186/1471-2458-11-295

**Published:** 2011-05-10

**Authors:** Anne G Wheaton, Geraldine S Perry, Daniel P Chapman, Lela R McKnight-Eily, Letitia R Presley-Cantrell, Janet B Croft

**Affiliations:** 1 Division of Adult and Community Health, National Center for Chronic Disease Prevention and Health Promotion, Centers for Disease Control and Prevention, 4770 Buford Highway NE, Mailstop K-67, Atlanta, GA 30041, USA

## Abstract

**Background:**

Over the past 50 years, the average sleep duration for adults in the United States has decreased while the prevalence of obesity and associated outcomes has increased. The objective of this study was to determine whether perceived insufficient sleep was associated with body mass index (BMI) in a national sample.

**Methods:**

We analyzed data from the 2008 Behavioral Risk Factor Surveillance System (BRFSS) survey (N = 384,541) in which respondents were asked, "During the past 30 days, for about how many days have you felt you did not get enough rest or sleep?" We divided respondents into six BMI categories and used multivariable linear regression and logistic regression analyses to assess the association between BMI categories and days of insufficient sleep after adjusting for sociodemographic variables, smoking, physical activity, and frequent mental distress.

**Results:**

Adjusted mean days of insufficient sleep ranged from 7.9 (95% confidence interval [CI]: 7.8, 8.0) days for people of normal weight to 10.5 (95% CI: 10.2, 10.9) days for those in the highest weight category (BMI ≥ 40). Days of perceived insufficient sleep followed a linear trend across BMI categories. The likelihood of reporting ≥14 days of insufficient sleep in the previous 30 days was higher for respondents in the highest weight category than for those who were normal weight (34.9% vs. 25.2%; adjusted odds ratio = 1.7 (95% CI: 1.5, 1.8]).

**Conclusion:**

Among U.S. adults, days of insufficient rest or sleep strongly correlated with BMI. Sleep sufficiency should be an important consideration in the assessment of the health of overweight and obese people and should be considered by developers of weight-reduction programs.

## 1. Background

Over the past 50 years, the prevalence of obesity among U.S. adults has nearly tripled from about 13% in 1960-1962 to 34% in 2007-2008 [1,2]. Obesity is a major risk factor for many chronic diseases including type 2 diabetes, cardiovascular diseases, cancer, and obstructive sleep apnea [3-6]. As the prevalence of obesity increased, the percentage of U.S. adults who reported an average of ≤6 hours of sleep per day also increased, from 22-23% in 1985 to nearly 30% in 2005-2007 [7,8]. Results from a recent analysis of Behavioral Risk Factor Surveillance System (BRFSS) data from 2008 showed that only 30% of U.S. adults felt they had received adequate sleep every night in the previous month and that more than 10% felt they had received inadequate sleep every night [9]. Causes of sleep loss other than sleep disorders include lifestyle and occupational factors [10]. Numerous cross-sectional studies have found an inverse relationship between sleep duration and body mass index (BMI) [11,12]; however, others have found a U-shaped association, with a lower BMI among people averaging 7-8 hours of sleep and a higher BMI among those with shorter and longer sleep durations [13-15]. These studies relied on subjective reports of sleep duration, but the CARDIA Sleep Study, which used an objective measure (wrist actigraphy), also found that BMI was inversely associated with sleep duration, as well as with sleep fragmentation [16]. Recent laboratory-based studies have demonstrated that chronic sleep restriction may contribute to obesity by decreasing levels of leptin and increasing levels of ghrelin, hormones involved in satiety and hunger-promotion, respectively [17,18].

Results of a previous analysis of 2002 BRFSS data from 18 states concerning perceived insufficient rest or sleep showed that the prevalence of obesity (defined as a BMI ≥ 30) was higher among respondents who reported ≥14 days of insufficient sleep in the previous 30 days than among those who reported <14 days [19]. To confirm and expand on these previous findings showing a positive association between multiple levels of BMI and perceived insufficient rest or sleep in a national sample, we analyzed 2008 BRFSS data and adjusted results for sociodemographic variables, as well as for smoking, physical activity, and frequent mental distress.

## 2. Methods

The BRFSS collects data through annual state-based telephone surveys of non-institutionalized U.S. civilians aged ≥18 years. The surveys are conducted in all 50 states, the District of Columbia, and three U.S. territories (Guam, Puerto Rico, and the Virgin Islands). As the BRFSS is a public-use dataset, this research was exempt from review by an institutional review board. The core questionnaire of the 2008 BRFSS survey, which was administered to all survey participants, included the following question, "During the past 30 days, for about how many days have you felt you did not get enough rest or sleep?"

Survey participants' BMI was calculated from their self-reported weight and height (weight [kg]/height [m^2^]); and their BMI-based weight classification was determined on the basis of National Heart, Lung, and Blood Institute criteria: underweight (BMI < 18.5), normal weight (BMI = 18.5-24.9), overweight (BMI = 25.0-29.9), obese class I (BMI = 30.0-34.9), obese class II (BMI = 35.0-39.9), and obese class III (BMI ≥ 40) [20]. Female survey participants were also asked whether they were currently pregnant.

In our analysis, we assessed the extent to which two measures of sleep (mean number of days of insufficient sleep and prevalence of ≥14 days of insufficient sleep) were associated with BMI category. We also assessed the extent to which these measures were associated with sex, age in years (18-24, 25-34, 35-44, 45-54, 55-64, and ≥65), race/ethnicity (non-Hispanic white, non-Hispanic black, Hispanic, and non-Hispanic multiracial/other), education level (less than high school graduate, high school graduate or GED recipient, some college, and college graduate), smoking status (current, former, and never), recent physical activity (yes or no), and frequent mental distress (yes or no). Current smoking was defined as having smoked at least 100 cigarettes in one's lifetime and now smoking on at least "some days." Former smoking was defined as having smoked at least 100 cigarettes in one's lifetime but not currently smoking. Never smoking was defined as not having smoked at least 100 cigarettes in one's lifetime. Any recent physical activity was defined on the basis of survey participants' response to the question, "During the past month, other than your regular job, did you participate in any physical activities or exercises such as running, calisthenics, golf, gardening, or walking for exercise?" Frequent mental distress was defined on the basis of participants' response to the question, "Now thinking about your mental health, which includes stress, depression, and problems with emotions, for how many days during the past 30 days was your mental health not good?" A response of ≥14 days in the previous 30 days indicated frequent mental distress.

We conducted all analyses using SAS-callable SUDAAN (version 10.0.0, Research Triangle Park, NC) to account for the complex sampling design of the BRFSS. We used multivariate linear regression analyses to calculate mean days of insufficient rest or sleep adjusted for sex, race/ethnicity, age, education level, smoking status, recent physical activity, and frequent mental distress. We used multivariate logistic regression analyses to calculate the proportion of respondents reporting ≥14 days of insufficient sleep during the previous 30 days by BMI category and odds ratios (ORs) for receiving ≥14 days of insufficient sleep by BMI category. All ORs were adjusted for sex, race/ethnicity, age, education level, smoking status, recent physical activity, and frequent mental distress.

## 3. Results

The median response rate to the 2008 BRFSS survey among all 50 states and the District of Columbia was 53.3% (35.8%-65.9%), based on Council of American Survey and Research Organizations (CASRO) guidelines, and the median cooperation rate was 75.0% (59.3%-87.8%). There were 414,509 respondents to the 2008 BRFSS survey. We excluded from our analysis those with missing data for age (n = 3,653), BMI (n = 18,677), or days of inadequate sleep (n = 7,060), as well as women who indicated they were pregnant, weren't sure, or did not respond to a question about their pregnancy status (n = 3,237). After these exclusions, our study sample consisted of 384,541 U.S. adults (92.8% of all 2008 BRFSS survey respondents).

Weighted population characteristics are shown in Table 1. Nearly one-fifth of people were current smokers, and one-quarter reported no leisure-time physical activity in the previous month. Approximately one-tenth reported frequent mental distress. Only 1.8% of the population was underweight, 35.1% was normal weight, 36.4% was overweight, 17.1% was obese class I, 6.1% was obese class II, and 3.5% was obese class III. Approximately 30% reported 0 days of insufficient sleep or rest, and 11.1% reported getting insufficient rest or sleep all of the previous 30 days.

**Table 1:** Characteristics of respondents to perceived insufficient sleep question: Behavioral Risk Factor Surveillance System Survey, 2008.

Characteristic	**n**^**1**^	**%**^**2**^
**Total**	384,541	100.0
**Sex**		
Men	150,407	50.5
Women	234,134	49.5
**Race/Ethnicity**		
White, non-Hispanic	305,289	68.9
Black, non-Hispanic	29,745	9.8
Hispanic	25,255	14.5
Other/multiracial^3^	21,051	6.8
**Age**		
18-24	12,794	12.2
25-34	35,636	17.9
35-44	57,994	19.1
45-54	79,775	19.3
55-64	82,897	14.5
65+	115,445	17.0
**Education Level**		
<High school diploma	36,522	10.9
High school diploma or GED	115,468	28.7
Some college	102,219	26.7
College graduate	129,838	33.8
**Smoker**		
Current	65,956	18.7
Former	114,800	24.6
Never	202,463	56.6
**Physical Activity in Past Month**		
No	104,238	25.0
Yes	279,924	75.0
**Frequent Mental Distress**^**4**^		
No	340,634	89.7
Yes	39,088	10.4
**BMI Category**^**5**^		
Underweight	6,253	1.8
Normal weight	131,009	35.1
Overweight	141,082	36.4
Obese class I	67,084	17.1
Obese class II	24,609	6.1
Obese class III	14,504	3.5
**Days of Insufficient Sleep**		
0 days	137,781	30.6
1-13 days	148,476	41.5
14-29 days	57,931	16.9
30 days	40,353	11.1

^1 ^Unweighted n's. Categories may not sum to survey total because of missing responses.

^2 ^Weighted percentage.

^3 ^Asian, Native Hawaiian or Other Pacific Islander, American Indian/Alaska Native, or multiracial.

^4 ^Frequent mental distress: Mental health, including stress, depression, and problems with emotions, was not good for ≥14 days in past 30 days.

^5 ^BMI categories: underweight (BMI < 18.5), normal weight (18.5 ≤ BMI < 25), overweight (25 ≤ BMI < 30), obese class I (30 ≤ BMI < 35), obese class II (35 ≤ BMI < 40), obese class III (BMI ≥ 40).

Among all adults, the adjusted mean number of insufficient days of sleep in the previous 30 days was 8.6 (95% CI: 8.5, 8.6) (Table 2). The mean number of days was higher among women than among men (9.1 and 8.0, respectively [p < 0.05]) and lower among Hispanics than among members of other racial/ethnic groups. In general, age was inversely associated with days of insufficient sleep, with adults aged 18-34 reporting the most days of insufficient sleep, and those aged 65 or older reporting the fewest. The mean number of days of insufficient sleep ranged from 8.3 days among individuals without a high school diploma to 8.8 days among those with some college; was higher among current smokers than among nonsmokers; and higher among people who reported no leisure-time physical activity in the previous month than among those who reported at least some such activity. The mean number of days of insufficient sleep was 15.6 days among adults who reported frequent mental distress, compared to 7.8 days for adults without frequent mental distress.

**Table 2:** Perceived insufficient sleep among U.S. adults, aged ≥18 years, by selected characteristics: BRFSS, 2008.

		Number of Days (in Past 30 Days)		≥14 Days of Insufficient Sleep in Past 30 Days			
	**n**^**1**^	**Mean**^**2**^	(95% CI)	**%**^**2**^	(95% CI)	**OR**^**2**^	(95% CI)
**Total**	384,541	8.6	(8.5-8.6)	27.9	(27.6-28.2)		
**Sex**							
Men	150,407	8.0	(7.9-8.1)	25.9	(25.4-26.3)	Referent	
Women	234,134	9.1	(9.0-9.2)	29.9	(29.5-30.4)	1.3	(1.2-1.3)
**Race/Ethnicity**							
White, non-Hispanic	305,289	8.9	(8.9-9.0)	29.2	(28.8-29.5)	Referent	
Black, non-Hispanic	29,745	8.5	(8.3-8.8)	27.3	(26.3-28.4)	0.9	(0.9-1.0)
Hispanic	25,255	6.9	(6.6-7.1)	22.3	(21.3-23.3)	0.7	(0.6-0.7)
Other/multiracial^3^	21,051	8.4	(8.1-8.7)	28.2	(26.9-29.5)	0.9	(0.9-1.0)
**Age**							
18-24	12,794	10.2	(9.9-10.5)	33.9	(32.5-35.4)	3.1	(2.8-3.3)
25-34	35,636	10.5	(10.3-10.7)	35.1	(34.2-36.0)	3.2	(3.1-3.4)
35-44	57,994	9.7	(9.6-9.9)	32.1	(31.4-32.8)	2.8	(2.7-2.9)
45-54	79,775	8.7	(8.6-8.8)	27.9	(27.3-28.4)	2.3	(2.2-2.4)
55-64	82,897	7.2	(7.1-7.3)	23.0	(22.5-23.6)	1.7	(1.6-1.8)
65+	115,445	5.0	(4.9-5.1)	15.4	(15.0-15.8)	Referent	
**Education Level**							
<High school diploma	36,522	8.3	(8.1-8.6)	27.3	(26.2-28.4)	1.0	(0.9-1.1)
High school diploma or GED	115,468	8.4	(8.2-8.5)	27.2	(26.7-27.8)	1.0	(0.9-1.0)
Some college	102,219	8.8	(8.7-9.0)	29.1	(28.6-29.7)	1.1	(1.0-1.1)
College graduate	129,838	8.6	(8.5-8.7)	27.5	(27.0-28.1)	Referent	
**Smoker**							
Current	65,956	9.9	(9.7-10.0)	32.7	(31.9-33.4)	1.4	(1.4-1.5)
Former	114,800	8.6	(8.5-8.7)	28.3	(27.7-28.9)	1.1	(1.1-1.2)
Never	202,463	8.1	(8.0-8.2)	26.0	(25.6-26.4)	Referent	
**Physical Activity in Past Month**							
No	104,238	9.6	(9.5-9.8)	32.2	(31.6-32.8)	1.4	(1.3-1.4)
Yes	279,924	8.2	(8.1-8.3)	26.4	(26.1-26.8)	Referent	
**Frequent Mental Distress**^**4**^							
No	340,634	7.8	(7.7-7.8)	24.5	(24.2-24.8)	Referent	
Yes	39,088	15.6	(15.4-15.8)	55.9	(54.8-56.9)	4.2	(4.0-4.4)
**BMI Category**^**5**^							
Underweight	6,253	8.6	(8.0-9.2)	27.6	(25.2-30.0)	1.1	(1.0-1.3)
Normal weight	131,009	7.9	(7.8-8.0)	25.2	(24.7-25.7)	Referent	
Overweight	141,082	8.5	(8.4-8.6)	27.6	(27.1-28.1)	1.1	(1.1-1.2)
Obese class I	67,084	9.3	(9.1-9.5)	30.8	(30.1-31.6)	1.4	(1.3-1.4)
Obese class II	24,609	9.7	(9.4-10.0)	32.3	(31.1-33.5)	1.5	(1.4-1.6)
Obese class III	14,504	10.5	(10.2-10.9)	34.9	(33.3-36.5)	1.7	(1.5-1.8)

^1 ^Unweighted n's.

^2 ^Weighted means, proportions, and odds ratios adjusted for all other variables in the table.

^3 ^Asian, Native Hawaiian or Other Pacific Islander, American Indian/Alaska Native, or multiracial.

^4 ^Frequent mental distress: Mental health, including stress, depression, and problems with emotions, was not good for ≥14 days in past 30 days.

^5 ^Underweight (BMI < 18.5), normal weight (18.5 ≤ BMI < 25), overweight (25 ≤ BMI < 30), obese class I (30 ≤ BMI < 35), obese class II (35 ≤ BMI < 40), obese class III (BMI ≥ 40).

Overall, 27.9% of adults reported ≥14 days of insufficient sleep in the previous 30 days (Table 2). The percentage who did so was higher among women than among men (29.9% vs. 25.9%, p < 0.05); lower among Hispanics than among members of the other racial/ethnic groups; lower among those aged 65 or older (15.4%) than among those aged 25-34 (35.1%); higher among those with some college education; lower among never smokers than among former smokers and current smokers (26.0% vs. 28.3% and 32.7%, respectively, p < 0.05); and higher among those who reported no leisure-time physical activity in the previous month than among those who reported some such activity (32.2% vs. 26.4%, p < 0.05). More than half of adults who reported frequent mental distress also reported ≥14 days of insufficient sleep in the previous 30 days, compared to about a quarter of adults without frequent mental distress.

When adjusted for sociodemographic characteristics, smoking status, recent physical activity, and frequent mental distress, the mean number of days of insufficient sleep ranged from 7.9 (95% CI, 7.8, 8.0) for people in the normal-weight category to 10.5 (95% CI, 10.2, 10.9) for those in the obese class III category. The mean number was also higher for people who were underweight (8.6 [95% CI, 8.0, 9.2]) than for those of normal weight. A quarter of normal-weight people reported ≥14 days of insufficient sleep in the previous month, compared with 34.9% of those in the obese class III category.

**Figure 1:**
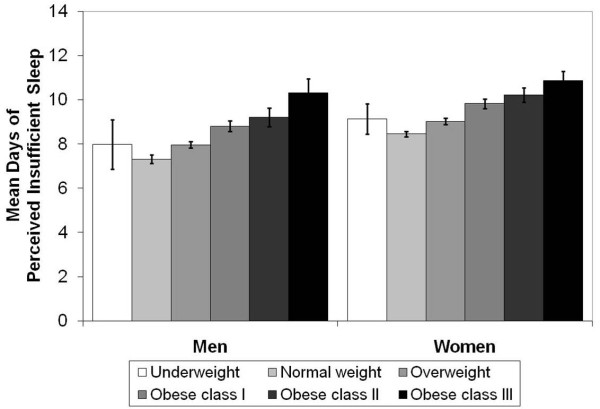
**Mean (±95% confidence intervals) days of perceived insufficient sleep in previous 30 days by BMI category and sex**. Means adjusted for race, age, education, smoking, recent physical activity, and frequent mental distress.

**Figure 2:**
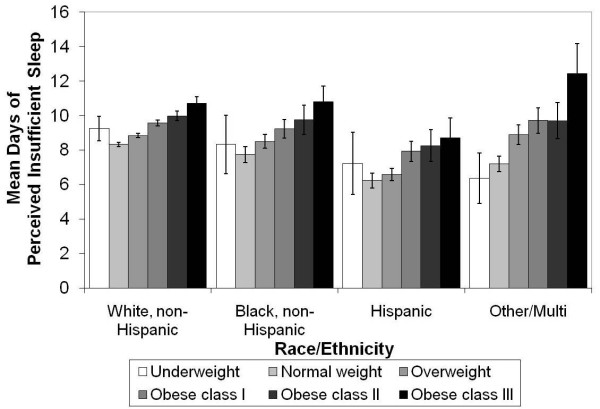
**Mean (±95% confidence intervals) days of perceived insufficient sleep in previous 30 days by BMI category and race/ethnicity**. Means adjusted for sex, age, education, smoking, recent physical activity, and frequent mental distress.

**Figure 3:**
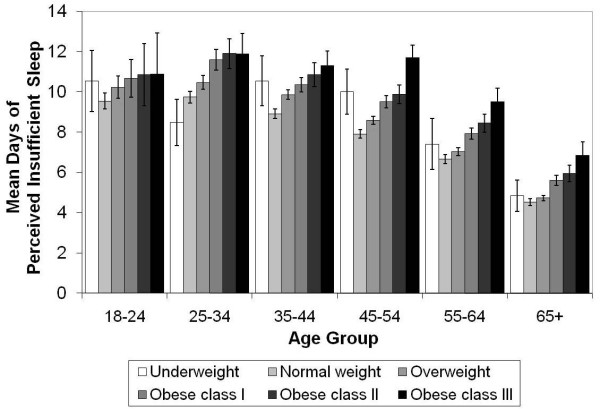
**Mean (±95% confidence intervals) days of perceived insufficient sleep in previous 30 days by BMI category and age**. Means adjusted for sex, race, education, smoking, recent physical activity, and frequent mental distress.

We observed a clear, positive gradient relationship between days of insufficient sleep and BMI-based weight categories from normal weight through obese class III among both men and women (Figure 1), all racial/ethnic groups (Figure 2), and all age groups (Figure 3). Although the number of days of insufficient sleep was generally higher among people in the underweight category than among those in the normal-weight category, the difference was not always statistically significant.

## 3. Discussion

Most studies that have assessed the association between BMI and sleep have used self-reported usual sleep duration as their sleep measure. As noted previously, results from many of these studies have shown a simple inverse relationship between sleep duration and BMI, whereas results from others have shown a U-shaped relationship, with a higher BMI associated with both short and long sleep durations [21-23]. Other study results have shown increased morbidity and mortality risk among people reporting either short or long sleep durations [24-27]. We detected a strong association between days of insufficient sleep and BMI category except among people classified as underweight. A likely explanation for this exception to our overall finding is that the prevalence of conditions that both cause weight loss and disrupt sleep, such as eating disorders, cancer, or other chronic diseases, may be higher in the underweight group.

Some study results have shown that the association between obesity (or BMI) and sleep duration differs by sex; however, the direction of the difference has not been consistent. For example, Kripke et al. found a negative association between sleep duration and BMI among men but a U-shaped relationship among women [28]; Cournot et al. reported that short sleep duration was associated with higher BMI among women but not among men [29]; and results of a study conducted in Hong Kong showed a negative association between sleep duration and BMI among men but not among women [30]. Longitudinal studies that have examined changes in weight with sleep duration or sleep problems have also been inconsistent. In a longitudinal study from Finland, sleep problems such as trouble falling asleep and trouble staying asleep were associated with major weight gain during a 5- to 7-year follow up among middle-aged women, but not men [31]. In contrast, short sleep duration was associated with weight gain at 1-year follow up in a large Japanese study among men, but not women [32]. Although we observed that women experienced more days of insufficient sleep than men, we saw a similar positive association between BMI and number of days of insufficient sleep among each.

We found that Hispanics had fewer days of insufficient sleep than blacks or whites. Results from the 2004-2007 National Health Interview Survey similarly showed that Mexican-Americans were more likely to experience long sleep duration than other race/ethnicities, although they also indicated that non-Hispanic blacks were more likely to experience both short and long sleep duration than were non-Hispanic whites [33]. In the CARDIA Sleep Study, time in bed, sleep duration, sleep latency (time between going to bed and falling asleep), and sleep efficiency (percentage of time in bed spent sleeping) varied by both race and sex: white women had the longest sleep duration, highest sleep efficiency, and shortest sleep latency, and black men had the shortest sleep duration, lowest sleep efficiency, and longest sleep latency [34].

Individuals aged 65 years or older reported the fewest days of insufficient sleep in the present study, and individuals aged 25-34 reported the most. In contrast, results of an analysis of data from the 2004-2007 National Health Interview Survey showed that older adults were more likely to report short and long sleep durations and that younger age was associated only with long sleep duration [33]. Among women enrolled as controls in the Collaborative Breast Cancer Study, age was negatively associated with hours of sleep [14]; the results of this study also showed that sleep duration was negatively associated with risk for obesity among women aged 50 or older as well as among those younger than 50 [14]. We similarly observed a positive association between days of insufficient sleep and BMI category across all age groups.

The prevalence of frequent insufficient sleep was higher among current and former smokers than among never smokers and among those who reported no recent leisure-time physical activity than among those who reported some such activity. This association between unhealthy behaviors and insufficient sleep is supported by previous analysis of National Health Interview Survey data that demonstrated that smoking and physical inactivity were more prevalent among individuals who slept less than 6 hours compared to those who slept 7 or 8 hours [35].

Whereas most studies of the relationship between BMI and sleep have used sleep duration as the measure for sleep, we used days of *perceived *insufficient rest or sleep. Comparison of the measure used in our study with self-reported sleep duration, snoring, and daytime sleepiness in the same population will enable us to refine our conclusions. One limitation to using sleep duration as the sole measure for sleep is that such a measure does not address the quality of sleep. Even people who sleep for a relatively long time may not get adequate quality sleep because their sleep is disrupted by sleep-disordered breathing, sleep disorders such as insomnia, the side-effects of various medications, or other unknown causes. Results of studies comparing self-reported sleep duration with objectively measured sleep duration have shown that people with poorer sleep quality reported sleeping for shorter periods than those with better sleep quality, although the measured sleep duration for the two groups was the same [36,37].

One recent study addressed the issue of sleep quality in an investigation of sleep duration and BMI. As part of the CARDIA Sleep Study, Lauderdale et al. [16] used wrist actigraphy to obtain objective measures of sleep duration and sleep fragmentation and also collected data on apnea symptoms, including snoring and tiredness. They found that both shorter sleep duration and greater sleep fragmentation were associated with higher BMI in unadjusted models, although adjustment for confounders (i.e., sociodemographic factors, smoking status, physical activity, and apnea risk factors) decreased the association between sleep duration and BMI and eliminated the association between sleep fragmentation and BMI, possibly because of the adjustment for snoring.

Our findings are subject to several limitations. First, the wording used in the insufficient sleep question is open to interpretation. For instance, respondents may interpret "enough" to mean at least a specific number of hours or rather sufficient time to awaken refreshed. The question also does not distinguish between "rest" and "sleep". Also, BRFSS data are collected through telephone surveys of the civilian, non-institutionalized population, therefore our findings are not generalizable to military personnel, institutionalized persons, and persons residing in households without landline telephones. Finally, the cross-sectional nature of the survey prevented us from attempting to determine the causal relationship between BMI and sleep. However, there is growing evidence that excess weight and insufficient and/or poor quality sleep may have a reciprocal causal relationship. Obesity has been shown to increase the risk for obstructive sleep apnea syndrome, a disorder characterized by frequent disruption of breathing during sleep caused by closure of the airways [38-40]. These abnormal breathing patterns result in disturbed sleep. Excess weight is strongly associated with the prevalence of sleep apnea, as well as with the frequency of disordered-breathing events and with oxygen desaturation [41,42]. In recent years, results from a few prospective cohort studies have shown weight gain to be associated with an increased risk of developing sleep apnea [38-40], and clinical trial results have shown weight loss among sleep apnea patients to be associated with a decrease in the severity of sleep apnea [43-45]. On the other hand, there is also evidence that chronic sleep disruption may alter appetite regulation by changing levels of hormones such as leptin and ghrelin [17,18]. One advantage to using BRFSS data, however, is that the large sample size of the BRFSS survey allowed us to assess the relationship between BMI and insufficient sleep for various subgroups based on sex, age, and race/ethnicity.

## 4. Conclusion

The results of our analysis, which showed a strong graded association between days of perceived insufficient sleep and weight status across *all *levels of BMI among U.S. adults, suggest that the possible effect of excess weight on sleep should be considered by developers of programs to address sleep disorders and that the possible effect of insufficient sleep on weight should be considered by developers of weight-reduction programs. Although sleep specialists commonly discuss with their patients how obesity may increase the risk for sleep disorders, they should also address the possibility that even smaller amounts of excess weight, as seen in overweight individuals, may be detrimental to their health and welfare.

## Competing interests

The authors declare that they have no competing interests.

## Authors' contributions

AW performed data analysis and drafted the manuscript. All authors contributed to the interpretation of the results and preparation and approval of the final manuscript.

## Pre-publication history

The pre-publication history for this paper can be accessed here:

http://www.biomedcentral.com/1471-2458/11/295/prepub
